# Impact of mitochondria on nitrite metabolism in HL-1 cardiomyocytes

**DOI:** 10.3389/fphys.2013.00101

**Published:** 2013-05-20

**Authors:** Peter Dungel, Andreas H. Teuschl, Asmita Banerjee, Jamile Paier-Pourani, Heinz Redl, Andrey V. Kozlov

**Affiliations:** ^1^Ludwig Boltzmann Institute for Experimental and Clinical Traumatology in AUVA research centreVienna, Austria; ^2^Department of Biochemical Engineering, University of Applied Sciences Technikum WienVienna, Austria

**Keywords:** nitrite, HL-1 cardiomyocytes, mitochondria, nitric oxide, hypoxia

## Abstract

Apart from ATP synthesis mitochondria have many other functions, one being nitrite reductase activity. Nitric oxide (NO) released from nitrite has been shown to protect the heart from ischemia/reperfusion (I/R) injury in a cGMP-dependent manner. However, the exact impact of mitochondria on the release of NO from nitrite in cardiomyocytes is not completely understood. Besides mitochondria, a number of non-mitochondrial metalloproteins have been suggested to facilitate this process. The aim of this study was to investigate the impact of mitochondria on the bioactivation of nitrite in HL-1 cardiomyocytes. The levels of nitrosyl complexes of hemoglobin (NO-Hb) and cGMP levels were measured by electron spin resonance spectroscopy and enzyme immunoassay. In addition the formation of free NO was determined by confocal microscopy as well as intracellular nitrite and S-nitrosothiols by chemoluminescence analysis. NO was released from nitrite in cell culture in an oxygen-dependent manner. Application of specific inhibitors of the respiratory chain, p450, NO synthases (NOS) and xanthine oxidoreductase (XOR) showed that all four enzymatic systems are involved in the release of NO, but more than 50% of NO is released via the mitochondrial pathway. Only NO released by mitochondria activated cGMP synthesis. Cardiomyocytes co-cultured with red blood cells (RBC) competed with RBC for nitrite, but free NO was detected only in HL-1 cells suggesting that RBC are not a source of NO in this model. Apart from activation of cGMP synthesis, NO formed in HL-1 cells diffused out of the cells and formed NO-Hb complexes. In addition nitrite was converted by HL-1 cells to S-nitrosyl complexes. In HL-1 cardiomyocytes, several enzymatic systems are involved in nitrite reduction to NO but only the mitochondrial pathway of NO release activates cGMP synthesis. Our data suggest that this pathway may be a key regulator of myocardial contractility especially under hypoxic conditions.

## Introduction

For decades nitrite and nitrate were thought to be the stable end products of nitric oxide (NO) produced by various isoforms of NO synthase (NOS). However, this concept became outdated when it was recognized that, under hypoxic conditions, nitrite can be reduced back to NO (Zweier et al., 1995; Lepore et al., 1999; Cosby et al., 2003; Vanin et al., 2007; van Faassen et al., 2009). Nitrite-derived NO creates a number of effects, for example increased levels of nitroso species, alters redox status and induces protein modifications (Dezfulian et al., 2007, 2009; Perlman et al., 2009). These effects may be involved in the cytoprotective effects of nitrite seen in models of local ischemia/reperfusion (I/R) injury in the heart, liver and brain (Lu et al., 2005; Jung et al., 2006; Bryan et al., 2008; Calvert and Lefer, 2009). Cytoprotection is attributed to the reduction of nitrite to NO and consequent activation of cGMP synthesis (Shiva and Gladwin, 2009), which leads to vasodilation and thus, better perfusion and increased oxygen supply (Wolin, 2009).

However, the mechanisms by which endogenous or exogenous nitrite is reduced to NO are still under discussion. One potential pathway occuring in blood is the reduction of nitrite by hemoglobin (Hb) in red blood cells (RBC). Cosby et al. suggested that nitrite-derived NO diffuses from the RBC into the smooth muscle cells of the vasculature and activates nitrite-mediated vasodilation (Cosby et al., 2003). Recently, however, it was suggested that not NO but another nitrogen species, N_2_O_3_, forms in RBCs diffuses into the tissues and induces vasodilation (Basu et al., 2007b). Lundberg et al. showed that the reduction of nitrite to NO depends on the predominant partial pressure of oxygen in the blood; lower oxygen levels facilitate NO release (Lundberg and Weitzberg, 2005). Hb seems to follow an allosteric regulation of nitrite reduction, showing the best nitrite reductase performance at 50% saturation with oxygen (Rassaf et al., 2007; van Faassen et al., 2009).

Another pathway of nitrite reduction other than the RBC pathway is mediated via parenchymal cells in tissues. Various intracellular enzymes have been identified to possess nitrite reductase activity, for example xanthine oxidoreductase (XOR) (Webb et al., 2008) and NOS (van Faassen et al., 2009). Also mitochondria can rapidly reduce nitrite to NO (Kozlov et al., 1999). It has been shown that NO reduced from nitrite by mitochondria in turn reversibly inhibits mitochondrial respiration (Nohl et al., 2000). However, it is not known whether or not this NO pool may escape from mitochondria and activate other signaling pathways.

Apart from regulation of vascular tonus, NO plays an important role in the cardiovascular system to maintain cardiac function (Rastaldo et al., 2007) via activation of cGMP synthesis in a dose-dependent manner. Under physiologic conditions endothelial and neuronal NOS play an important role in cardiac function. The inhibition of NO synthesis catalyzed by NOS under normoxic conditions induces cardiac remodeling (hypertrophy), which is independent of the systemic hemodynamic effects of NO (Sanada et al., 2003). Nitrite represents a NOS-independent NO-source that becomes effective under hypoxic conditions. Nitrite, however, represents a NOS-independent NO-source that becomes effective under hypoxic conditions like I/R. Previously it was shown that the reduction of nitrite to NO in heart homogenate is nearly fully inhibited by myxothiazol, a specific inhibitor of the mitochondrial respiratory chain at complex III (Kozlov et al., 2005). However, the impact of this pathway on the bioactivation of nitrite in cardiomyocytes has not been addressed in the literature.

The aim of this study was to clarify the impact of mitochondria on nitrite reduction in HL-1 cardiomyocytes under hypoxic conditions.

## Materials and methods

### Preparation of HL-1 cells and RBC

HL-1 cardiomyocytes were used in this study. Cells were cultured under a 5% CO_2_ atmosphere in Claycomb medium (Sigma, St. Louis, USA) supplemented with 10% fetal bovine serum (Lonza, Basel, Switzerland), 4 mM L-glutamine (Sigma, St. Louis, USA), 100 U/ml penicillin (Sigma, St. Louis, USA), 100 μg/ml streptomycin (Sigma, St. Louis, USA), and 100 μM norepinephrine (Sigma, St. Louis, USA). Cells were seeded in 24-well cell culture plates precoated with 25 μg/ml fibronectin and 0.02% gelatin solution and cultivated until confluent.

To isolate human RBC, whole blood was centrifuged at 1600 g for 10 min, plasma and buffy-coat discarded and the remaining RBC washed twice with phosphate buffered saline (PBS). RBC were then diluted with PBS accordingly and counted on a Cell-Dyn 3700 (Abbott, Switzerland) before use.

### Experimental setup

#### Determination of nitrite-derived NO-release from HL-1 cells

Experiments were performed in a gas-tight glove box, which allowed to study nitrite reduction under various oxygen concentrations (from 21% to <1% O_2_). All solutions were deoxygenated with nitrogen prior to experiment. Twenty-four-well cell culture plates with confluent HL-1 cells (approx. 250,000 cells/well) were transferred into the glove box containing nitrogen atmosphere. In selected experiments HL-1 cells had been preincubated under 21% oxygen for 1 h with either 10 μM myxothiazol, 10 mM potassium cynide, 1 mM allopurinol, 300 μM N-nitro-L-arginine methyl ester (L-NAME) or 500 μM methyrapone. The following procedures were performed inside the glove box: to measure NO-Hb, the cell medium was exchanged with diluted RBC in PBS and cells treated with 50 μM nitrite or PBS as control. For analysis of cGMP synthesis medium was exchanged with PBS, followed by addition of 50 μM nitrite or PBS. PBS contained 1 mM of the phosphodiesterase inhibitor (MacArthur et al., 2007) isobutylmethylxanthin (IBMX) to prevent cGMP degradation. Cells were then incubated under hypoxic conditions on a shaker at 37°C for 1 h. Afterwards, the supernatant was withdrawn for analyses of NO-Hb and cGMP, the remaining cells were lysed by sonification in 500 μl lysis reagent provided with the enzyme immunoassay for detection of cGMP (GE Healthcare, Fairfield, USA) and the lysates stored for quantification of intracellular nitrite and nitroso species. Protein content of the lysates was determined for normalization by BCA protein assay (Pierce, Rockford, USA). In selected experiments NO-gas was used as a positive control. Physiological saline was purged with NO gas (Messer, Austria) for 15 min. NO concentration was determined with a NO electrode (World Precision Instruments Ltd., USA) and was 1 mM in average.

### EPR spectroscopy

The EPR spectra from frozen samples were recorded in a Miniscope MS 2000 (Magnettech Ltd., Berlin, Germany) at liquid nitrogen temperature with the following settings: microwave frequency 9.429 GHz, microwave power 30 mW, modulation frequency 100 kHz and modulation amplitude 6 G.

### Confocal microscopy

The detection of NO production in living cells was performed using the NO-sensitive fluorescent dye 4-Amino-5-methylamino-2′,7′-difluorofluorescein diacetate (DAF-FM diacetate; Molecular Probes, Carlsbad, CA). DAF-FM diacetate is nitrosated by NO and is a reasonable indication for NO. HL-1 cells or RBC were loaded with 10 μ M DAF-FM diacetate and incubated for 30 min at 37°C. Afterwards, 50 μ M of NaNO2 were added and cells were incubated for another 30 min at 37°C under anaerobic conditions in an incubator at <1% oxygen. Imaging was performed with an inverted confocal microscope (LSM 510, Zeiss, Germany) with excitation/emission maxima of 495/515 nm, 63× oil immersion objective and 2.0 scan zoom.

### Analysis of nitrite and nitroso species

Nitrite and nitroso species in cell lysates were measured using a triiodide-based reductive chemiluminescence method with a NO analyzer (model 280, Seivers, Boulder, USA). To determine the levels of nitrite and nitroso species two aliquots of each sample were analyzed, as previously described (MacArthur et al., 2007). The first aliquot was directly injected into the triiodide reagent to measure nitrite, the second was incubated for 10 min with acidified sulfanilamide (0.5% vol:vol) to eliminate nitrite before injected into the triiodide reagent. Since the second measurement represents the signal from nitroso species, the comparison of both peaks provides an accurate method for measuring nitrite.

### cGMP measurement

cGMP levels in HL-1 cells were measured by enzyme immunoassay (cGMP EIA; GE Healthcare, Fairfield, USA) according to protocol 3 of the manufacturer's instructions. Briefly, cell culture supernatants were aspirated and adherent cells lysed in the lysis reagent provided. Samples were acetylated prior to performing the assay. Optical density (405 nm) was measured by a plate reader (Tecan, Männedorf, Switzerland) and the concentration of cGMP was calculated from a standard curve produced from serial dilutions of acetylated cGMP solutions. All standards and samples were assayed in duplicate.

### Data presentation and statistics

Data represent mean ± SD. Significance was calculated using One-Way ANOVA test with *post-hoc* LSD (least significant difference) test.

## Results

**Figure 1:**
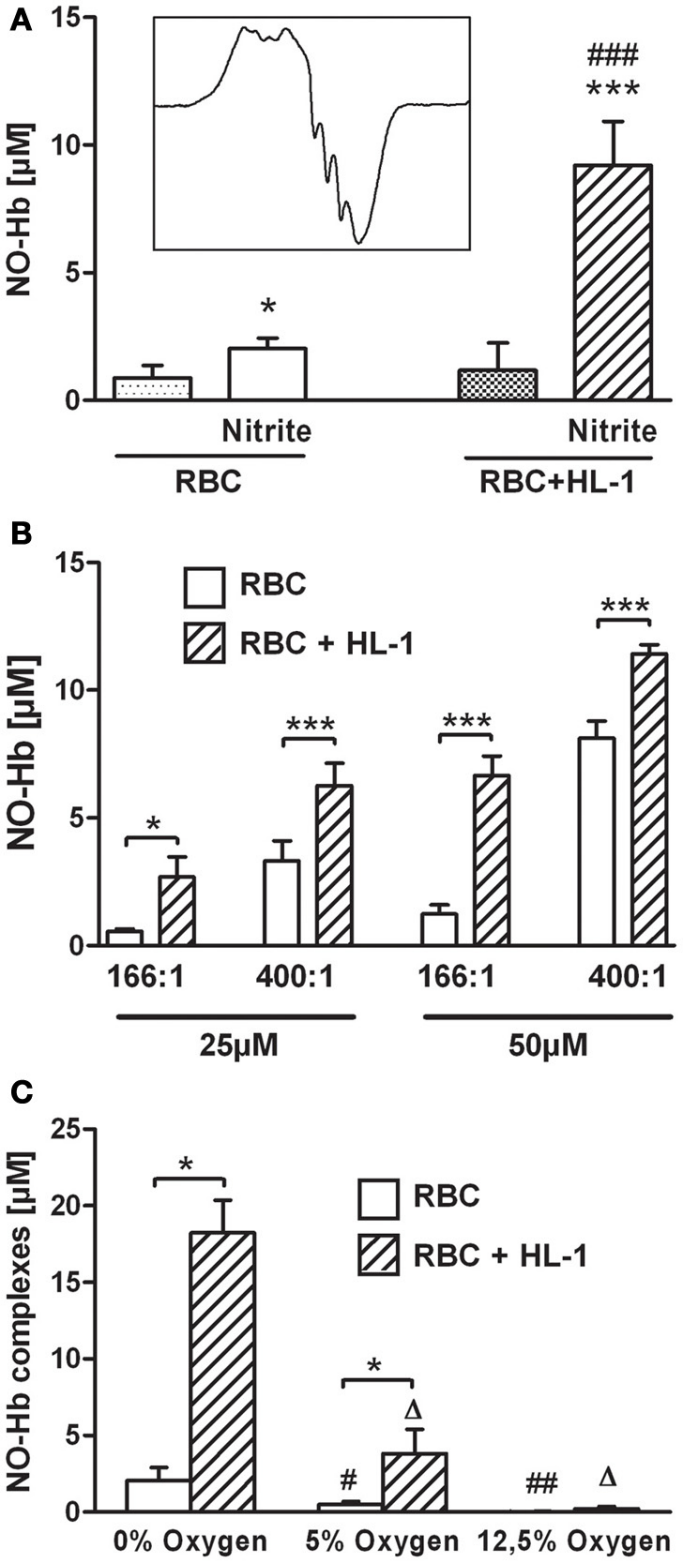
**(A)** NO production from RBC and in co-culture with HL-1 cells under hypoxic conditions with and without 50 μM nitrite. **(A)** Both RBC and HL-1 cells are capable of nitrite reduction. Left set of bars shows the RBC-mediated release of NO from nitrite. Right set of bars represents data from co-culture of RBC with HL-cells in a RBC:HL-1 ratio of 166:1 (^*^p < 0.005, ^***^p < 0.001 compared to the respective group without nitrite, ^###^*p* < 0.001, compared to RBC with nitrite). Inset in 1 **(A)** shows typical EPR spectrum of NO-Hb complexes. **(B)** The relative contribution of both pathways depends on both nitrite concentrations and the RBC:HL-1 ratio. A ratio of 166:1 and a nitrite concentration of 50 μM were chosen for all other experiments as these conditions showed the highest difference inNO-signals with and without HL-1 cells. ^*^p < 0.05, ^***^p < 0.001 RBC vs. RBC + HL-1 of the same group. **(C)** Oxygen-dependence of NO-Hb signal. RBC and in co-culture with HL-1 cells were incubated with 50 μM nitrite under various oxygen concentrations. ^*^p < 0.001, NO-Hb signals with and without HL-1 cells. ^Δ^*p* < 0.001, compared to RBC + HL-1 at 0% oxygen. ^#,##^*p* < 0.05 and *p* < 0.01, respectively, compared to RBC at 0% oxygen.

**Figure 2:**
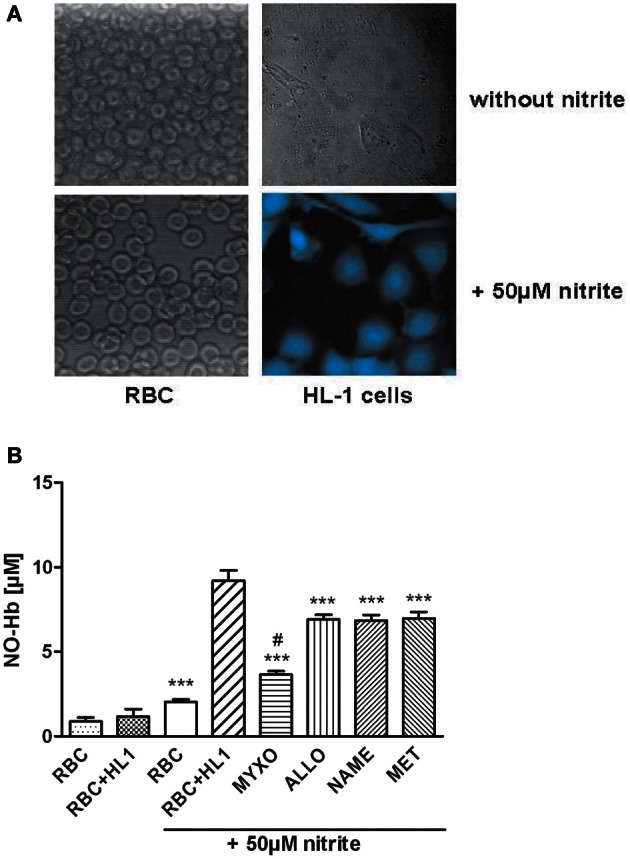
**(A)** Analysis of free NO using a NO-specific dye and confocal microscopy. HL-1 cells or RBC were loaded with 10 μM DAF-FM and incubated for about 30 min at 37°C. Afterwards, 50 μ M of NaNO_2_ were added and cells were incubated for another 30 min at 37°C under anaerobic conditions. Free NO could only be detected in HL-1 cells but not in RBC. **(B)** Influence of various inhibitors on NO-Hb signals of RBC in co-culture with HL-1 cells determined by EPR analysis. Myxothiazol, an inhibitor of complex III of the respiratory chain, reduced NO-signal by 60%, while all inhibitors reduced NO-Hb levels by approx. 25%. ^***^p < 0.001, compared to HL-1 cells incubated with 50 μM nitrite. ^#^*p* < 0.05, compared to RBC incubated with 50 μM nitrite.

Under hypoxic conditions NO reacts with Hb yielding nitrosyl complexes of hemoglobin (NO-Hb) with characteristic electron spin resonance spectra shown in the inset of Figure 1A. The baseline level of NO-Hb in RBC was doubled when RBC were incubated with nitrite, showing that RBC are well able to convert nitrite to NO. Co-culture with HL-1 cells led to a further significant increase in NO-Hb levels, indicating the significant portion of NO derived from HL-1 cells (Figure 1A). It also clearly demonstrated that the NO formed in HL-1 cells is released from the cells. Variation of the RBC:HL-1 ratio shows the relative contribution of RBC and HL-1 cells to NO formation (Figure 1B). A RBC:HL-1 ratio of 166:1 was chosen as it showed the highest difference between RBC and HL-1 derived NO. NO-Hb formation in both RBC and HL-1 cells was dependent on the partial pressure of oxygen (Figure 1C). The provision of free available NO, however, depends predominantly on parenchymal cells. The incubation of HL-1 cells with nitrite led to an increase in intracellular NO levels as revealed by confocal microscopy using the NO specific indicator dye DAF-2DA. In contrast, free NO was not detected in RBC as the fluorescence of RBC incubated with nitrite did not change compared to untreated control (Figure 2A).

Logically, the NO formed in cardiomyocytes has two major functions. A portion of NO diffusing out of cardiomyocytes may contribute to the regulation of vascular tonus and another portion may activate cGMP synthesis regulating myocardial contractility. The release of NO was determined by the formation of NO-Hb complexes in RBC co-cultured with HL-1 cells and cGMP levels were determined directly in HL-1 cells. To clarify the origin of nitrite-derived NO contributing to the formation of NO-Hb and cGMP synthesis, HL-cells were preincubated with various specific inhibitors, allowing definition of the impact of the respective enzymes. Regarding NO release and NO-Hb formation, all inhibitors used decreased the NO-Hb signal. However, the mitochondrial inhibitor myxothiazol had the most prominent effect, decreasing NO-Hb levels by 60%. Allopurinol, L-NAME and methyrapone, inhibitors of XOR, NOS and cytochrome P450, respectively, contributed to the formation of NO-Hb, although significantly less than mitochondria (Figure 2B).

**Figure 3:**
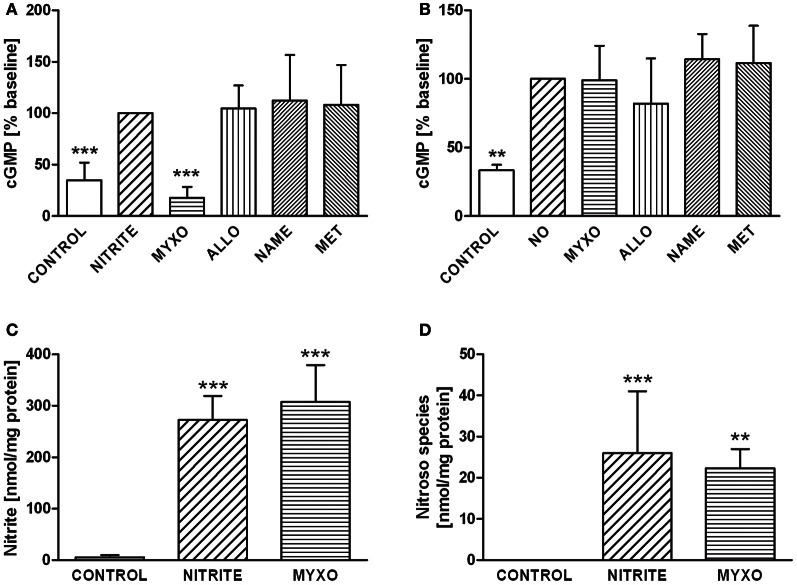
**(A)** GMP levels in HL-1 incubated with 50 μM nitrite under hypoxic conditions. cGMP production was completely inhibited by myxothiazol, suggesting that NO derived from nitrite reduction in mitochondria is predominately responsible for cGMP synthesis. Controls are untreated HL-1 cells, Nitrite indicates HL-1 cells treated with 50 μM nitrite, all other groups were treated with nitrite and with one of the following inhibitors: Myxo, Myxothiazol; Allo, Allopurinol; NAME, N-nitro-L-arginine methyl ester; Met, Methyrapone. **(B)** cGMP levels in HL-1 cells incubated with 50 μM NO under hypoxic conditions. GC activation by NO-gas was not influenced by either myxothiazol or any other inhibitor, demonstrating that nitrite reduction via mitochondria is predominately responsible for cGMP synthesis. Controls are untreated HL-1 cells, NO indicates HL-1 cells treated with 50 μM nitritc oxide, all other groups were treated with NO and with one of the following inhibitors: Myxo, Myxothiazol; Allo, Allopurinol; NAME, N-nitro-L-arginine methyl ester; Met, Methyrapone. ^**^p < 0.01, ^***^p < 0.001. Intracellular levels of nitrite **(C)** and nitroso species **(D)** in HL-1 cells. Controls are untreated HL-1 cells, Nitrite indicates HL-1 cells treated with 50 μM nitrite, Myxo represents HL-1 cells incubated with 50 μM nitrite preincubated with myxothiazol. Cell lysates were analysed by a Sievers NOA280i chemiluminescence analyser using the tri-iodide method to determine nitrite. For analysis of nitroso species a second sample was treated with acidified sulfanilamide to eliminate nitrite. ^***^p < 0.001 vs. control.

The impact of these enzymes on nitrite-dependent cGMP formation was investigated in a similar way. cGMP synthesis in HL-1 cells stimulated by nitrite-derived NO was fully prevented by myxothiazol while other inhibitors had no effect (Figure 3A). NO gas-saturated saline, used as a positive control, led to significant increase in cGMP production. However, NO-mediated guanylyl cyclase (GC) activation was influenced by neither myxothiazol nor any of the other inhibitors (Figure 3B).

Chemoluminescence analysis showed that incubation of HL-1 cells with 50 μM nitrite led to a drastic increase in intracellular nitrite (Figure 3C) and nitroso species (Figure 3D) levels. In contrast to NO-Hb and cGMP, nitroso species formation was not sensitive to myxothiazol.

## Discussion

In the past years it has been demonstrated that nitrite represents a storage pool of NO which can be activated, especially under hypoxic conditions. As nitrite reduction occurs both in blood Hb and tissue, we investigated the process of exogenous nitrite reduction in monocultures of HL-1 cells and RBC and in a co-culture of these two cell types. Since Hb is itself a nitrite reductase, RBC were incubated with and without nitrite to determine the RBC-dependent nitrite reduction. The free radical NO binds efficiently to deoxy-Hb in RBC, yielding the characteristic triplet spectrum of this NO-Hb complex. The formation of NO-Hb complexes in RBC incubated with nitrite reflects diffusion of nitrite into RBC and it reduction to NO. It has been demonstrated in the literature that nitrite reduction is oxygen-dependent. Figure 1C shows NO-Hb signals at anaerobic conditions as well as at oxygen concentrations typical for arterial (12.5% O_2_) and venous (5% O_2_) blood. In both RBC and co-culture with HL-1 cells, NO-Hb signals are reduced with a decrease in oxygen. There are two explanations for this finding: first, activities of nitrite reductases decrease with increases in oxygen or, second, Hb becomes oxygenated and reacts with NO to form methemoglobin rather than NO-Hb. In any case, anaerobic conditions yield the highest amounts of nitrite-derived NO and thus, further experiments were performed under anaerobic conditions. In cardiomyocytes, the ability of nitrite to diffuse into HL-1 cells was confirmed directly by determination of intracellular nitrite levels in HL-1 cell lysates (Figure 3D).

It has previously been shown that NO diffuses from diverse parenchymal cells into the blood to form NO-Hb complexes in RBC, but NO bound to Hb does not diffuse back to parenchymal cells (Kozlov et al., 2001). Consequently, we assumed that determination of NO-Hb complexes in our model is a measure of total NO-production in RBC plus HL-1 cells. In fact, addition of HL-1 cells to a RBC suspension increased nitrite mediated NO-Hb formation due to an additional portion of NO reduced in HL-1 cells. An increase in the RBC:HL-1 ratio results in an increase of the NO portion formed in HL-1 cells and reduced the NO portion coming from RBC. This shows that both RBC- and HL-1 mediated pathways have a distinguishable contribution to total NO production determined by NO-Hb levels. Also, the difference in NO-Hb levels between coculture of HL-1 cells and Hb and Hb alone is the NO formed in HL-1 cells. In this experimental model we found that free NO is only detectable in cardiomyocytes but not in RBC. This was shown by confocal microscopy combined with using the fluorescent dye DAF used to determine free NO in RBC and HL-1 cells upon treatment with nitrite. Increased DAF fluorescence was clearly detected in HL-1 cells but not in RBC, suggesting that free NO only occurs in HL-1 cells. This suggests that NO is tightly bound to Hb inside RBC. The lack of free NO in RBC seems to be in contradiction to the fact that RBC-mediated nitrite reduction contributes to vasodilation. However, this contradiction was satisfactorily explained in the recent publication of Basu et al. who suggested that N_2_O_3_, not NO, is formed in RBC and is the active mediator of vasodilation (Basu et al., 2007a).

The intracellular mechanisms of nitrite bioactivation in HL-1 cells were investigated by using inhibitors of various enzymes with distinct nitrite reductase activity. Specific inhibitors for XOR, complex III of the mitochondrial electron transport chain, cytochrome P450 and NOS were used. Cells were incubated with inhibitors prior to the addition of RBC which served as NO detection reagent. Thus, the effects of these inhibitors on the NO-Hb signal were only HL-1 dependent. All inhibitors reduced NO release, seen as lowered NO-Hb levels, but to various extents. The mitochondria-specific inhibitor myxothiazol decreased NO signal by 60%. This is in line with the fact that mitochondria contribute to nitrite reductase activity in heart homogenates by approx. 80% (Kozlov et al., 2003). However, NO-Hb levels were not reduced to baseline levels of untreated cells, suggesting that there are additional pathway(s) of NO production. As allopurinol, NAME and methyrapone reduced NO-signals by 25% each, this indicates other active mechanisms. According to Dr. Claycomb, who established the HL-1 cell line, these cells should contain myoglobin (Mb) comparable to primary cardiomyocytes (private communication). Mb is a potent nitrite reductase in the heart (Shiva et al., 2007) whose impact was not investigated in this study. However, Shiva et al. used ferricyanide to oxidize Mb in heart homogenate which led to decreased NO production. Ferricyanide is an unspecific one electron acceptor interacting also with the respiratory chain of mitochondria (Krasnikov et al., 1997). Thus, both nitrite and ferricyanide are reduced accepting electrons from the respiratory chain. Consequently, the effects of ferricyanide may be due to the inhibition of nitrite reduction by the respiratory chain. Thus, ferricyanide might not be suitable to distinguish between Mb-mediated and mitochondria-mediated nitrite reduction. On the other hand there are specific inhibitors of the mitochondrial respiratory chain, which do not react with Mb, for instance myxothiazol. In our previous publication we have shown that myxothiazol nearly completely inhibits nitrite reduction to NO in heart homogenate. This suggests that mitochondria are responsible for nitrite reduction in heart homogenate.

Another situation is demonstrated in the paper of Totzeck et al. (2012). They showed that Mb in SMC is involved in nitrite reduction and vasodilation. This suggests that the data from heart homogenate reflect nitrite reduction in cardiomyocytes (the mayor cell type contributing to heart homogenate), which is likely relevant for the regulation of myocardial contractility and Mb in SMC for vascular tonus.

Beside Mb, several studies showed that XOR also is an important nitrite reductase in the heart. McNulty et al. showed that in RBC-free heart tissue, nitrite consumption could be blocked by 40% by allopurinol (McNulty et al., 2008) but no mitochondria-specific inhibitor was tested in the study. One also has to keep in mind that allopurinol acts as an antioxidant as well, thus also inhibiting intracellular oxidative processes. Baker et al. demonstrated that both oxypuinol and diphenyleneiodonium (DPI), a non-specific flavoprotein inhibitor, inhibited nitrite-mediated cardioprotection (Baker et al., 2007). As DPI also inhibits mitochondrial complex I (Li and Trush, 1998), the reduction in cytoprotection might be due to reduced nitrite bioactivation by mitochondria. Irrespectively of the exact mechanism of allopurinol, we show that, in our model, mitochondria are the major contributor to the generation of diffusible NO.

Although mitochondria were only partially responsible for the generation of diffusible NO, they were the only NO source activating cGMP synthesis; myxothiazol completely inhibited cGMP synthesis and other inhibitors had no effect. This finding suggests that NO by mitochondria-mediated nitrite reduction is predominately responsible for cGMP synthesis in HL-1 cells. Zielinska et al. demonstrated that fluoroactetate, a metabolic inhibitor specifically affecting astrocytic mitochondria, inhibited cGMP synthesis by about 50% (Zielinska et al., 2007). An intracellular association of mitochondria and GC could explain that cGMP synthesis, but not NO-Hb signals, were completely blocked by mitochondrial inhibition.

To exclude the possibility that myxothiazol inhibited not only nitrite reduction, but also NO-mediated cGMP synthesis, we used pure NO as a control. GC activation by NO-gas was not influenced by either myxothiazol or any other inhibitor, showing the predominant contribution of mitochondria to nitrite bioactivation in cardiomyocytes under hypoxic conditions. These data suggest that the pathway responsible for stimulation of cGMP synthesis in cardiomyocytes was exclusively mitochondria-dependent. In addition, it has been shown that myxothiazol can also induce superoxide production in the mitochondrial matrix. Myxothiazol-derived superoxide may cause oxidation of NO produced in the mitochondria. However, myxothiazol did not inhibit cGMP production in NO-gas treated cells. These data may indicate that the nitrite reduction in the electron transfer chain occurs downstream of the myxothiazol binding site. This could point to complex IV, cytochrome c oxidase, which has recently been identified as a nitrite reductase (Castello et al., 2006).

Since NO has been cited to inhibit mitochondrial respiration nitrite reduction via complex IV the inhibition of this complex would initiate auto-regulation. Under hypoxic conditions nitrite reduction would continue until critical levels of NO will be reached to block the reaction at the same site.

We assume that this mitochondria-dependent increase in cGMP levels contributes to the regulation of myocardial contractility under hypoxic conditions and to cytoprotection (Cauwels et al., 2009). In contrast, formation of nitroso species upon nitrite treatment was not affected by myxothiazol. This suggests that nitroso compounds are formed from nitrite, not from NO within HL-1 cells.

In conclusion, in this study we showed *in vitro* that both RBC and HL-1 cardiomyocytes mediated the reduction of nitrite to NO under hypoxic conditions. RBC-mediated nitrite reduction did not contribute to cGMP synthesis in HL-1 cells. cGMP production in cardiomyocytes was completely inhibited by myxothiazol, a specific inhibitor of mitochondria, while inhibitors of XOR, NOS, and p450 had no effect. The effect of myxothiasol was not due to inhibition of cGMP synthesis itself, suggesting that mitochondria trigger nitrite-mediated cGMP synthesis in cardiomyocytes. Our data suggest that this pathway may be a key regulator of myocardial contractility, especially under hypoxic conditions.

### Conflict of interest statement

The authors declare that the research was conducted in the absence of any commercial or financial relationships that could be construed as a potential conflict of interest.
